# Retraction: Disruption of Growth Hormone Receptor Prevents Calorie Restriction from Improving Insulin Action and Longevity

**DOI:** 10.1371/journal.pone.0301671

**Published:** 2024-03-28

**Authors:** The PLOS ONE Editors

Following the publication of this article [1], concerns were raised regarding multiple figures. Specifically,

- In Fig 2C, the bands in lanes 5 and 6 appear similar to those in lanes 7 and 8, respectively. The corresponding author stated that this panel was spliced between lanes 2/3, 4/5, and 6/7, and acknowledged that the indicated bands appear similar but they do not believe they are identical.
- In Fig 3C, when color levels are adjusted, there appear to be vertical discontinuities suggestive of splice lines between lanes 4 and 5, and between lanes 6 and 7.
- In Fig 4F, there appears to be a vertical discontinuity between lanes 2 and 3. The corresponding author stated that they did not think this panel was spliced.
- During discussions, the corresponding author confirmed that the western blots in Figs 4C and 4G were spliced between lanes 2/3, 4/5, and 6/7.
- The legend for Figs 4C, F and G describes the use of liver homogenate, however the labels within the figure and the text describe these figures as presenting results from muscle. The corresponding author clarified that the figure legend is incorrect.

The corresponding author informed the journal that the first author (MSB) is deceased, and that due to this and because the experiments were performed 15–19 years ago, it was not possible to locate and accurately identify all the primary data for this article. For the uncropped western blot images that were located, the corresponding author acknowledged that labelling of the tissue types and samples presented in these images was missing or inconsistent, and that it was therefore not possible to fully confirm the content of these images. They stated that quantitative data related to the longevity, body weight and blood glucose results in this article remain available.

The uncropped images provided for Figs 2C, 4C, 4F and 4G do not appear to match the published panels based on editorial assessment, and in the absence of complete information about the samples presented in these images, they cannot be considered to support the published results. Therefore, the editors do not consider these images sufficient to resolve the concerns in these figures.

In light of the concerns affecting multiple figure panels that question the reliability of these data, and in the absence of primary data for the affected figures, the *PLOS ONE* Editors retract this article.

FPD, JSR, KAAR, RW, MMM, JJK, and AB did not agree with the retraction and stand by the article’s findings. OA, AS, and JP either did not respond directly or could not be reached. MSB is deceased.
